# Optimising opioid substitution therapy in the prison environment

**DOI:** 10.1108/IJPH-12-2017-0061

**Published:** 2019-12-05

**Authors:** Farrukh Alam, Nat Wright, Paul Roberts, Sunny Dhadley, Joanne Townley, Russell Webster

**Affiliations:** 1 Central and North West London NHS Foundation Trust, London, UK; 2 Transform Research Alliance, Leeds, UK; 3 HM Inspectorate of Prisons, London, UK; 4 Wolverhampton Volunteer Sector Council, Wolverhampton, UK; 5 Pathways to Recovery, Warrington, UK; 6 London, UK

**Keywords:** Health in prison, Prison, Drug abuse, Drug dependence, Opioid substitution therapy, Prison medicine

## Abstract

**Purpose:**

The purpose of this paper is to examine the current provision of opioid substitution therapy (OST) during and immediately following release from detention in prisons in England and Wales.

**Design/methodology/approach:**

A group of experts was convened to comment on current practices and to make recommendations for improving OST management in prison. Current practices were previously assessed using an online survey and a focus group with experience of OST in prison (Webster, 2017).

**Findings:**

Disruption to the management of addiction and reduced treatment choice for OST adversely influences adequate provision of OST in prison. A key concern was the routine diversion of opiate substitutes to other prisoners. The new controlled drug formulations were considered a positive development to ensure streamlined and efficient OST administration. The following patient populations were identified as having concerns beyond their opioid use, and therefore require additional considerations in prison: older people with comorbidities and complex treatment needs; women who have experienced trauma and have childcare issues; and those with existing mental health needs requiring effective understanding and treatment in prison.

**Originality/value:**

Integration of clinical and psychosocial services would enable a joint care plan to be tailored for each individual with opioid dependence and include options for detoxification or maintenance treatment. This would better enable those struggling with opioid use to make informed choices concerning their care during incarceration and for the period immediately following their release. Improvements in coordination of OST would facilitate inclusion of strategies to further streamline this process for the benefit of prisoners and prison staff.

## The current delivery of OST in prisons shows inconsistencies and is often carried out in isolation from routine clinical assessments

A recent Freedom of Information Request (2017) revealed that over 22,500 prisoners received opioid substitution therapy (OST) during the 2016 calendar year; an average of 205 individuals per prison. An online survey of 102 opiate-using detainees across the UK assessed the real-life access to OST by prisoners during their previous detention period (Webster, 2017).

The survey employed two main methods to gain service-user views. First, an online survey was completed by 102 opiate users who had been in an English or Welsh prison in the previous two years. Opiate-using prisoners were identified and recruited by ten peer researchers trained, supervised and supported by the Revolving Doors Agency. Researchers were based throughout England and Wales and each was set a target of recruiting ten survey respondents. Peer researchers received a payment of £100 each for recruiting survey respondents. Analysis of this survey data informed the second stage of the research study; a focus group comprising nine individuals with recent experience of OST in prison was convened by the Revolving Doors Agency and held on 10 May 2017. The focus group was structured to allow respondents to give detailed first-hand accounts of their experiences of seeking OST in prison and on their release.

Using these methods, the survey asked whether respondents wanted and were able to access OST in prison and on release, and how easy or not this access was. Respondents were also asked to suggest improvements to the current provision.

Survey respondents were first asked whether they wanted medication for their opiate dependency; whether they wanted methadone or buprenorphine; and whether they wanted to be on maintenance or withdrawal prescription. In total, 95 per cent of respondents did want medication, with a majority of respondents (49 per cent) wanting a maintenance prescription vs a withdrawal prescription (43 per cent) and also preferring methadone (61 per cent) to buprenorphine (31 per cent).

Respondents were then asked: “If you wanted medication, did you get it?” and were given a choice of five options that reflected ease of access:

1. Yes – I was offered medication.
2. Yes – I asked for medication and got it easily.
3. Yes – I asked for medication and had to work hard to get it.
4. Yes – I got some medication, but not what (or as much) as I wanted.
5. No – I did not get medication.

The vast majority of respondents (88/94=94 per cent) received at least some medication with varying degrees of ease of access.

**Figure 1:**
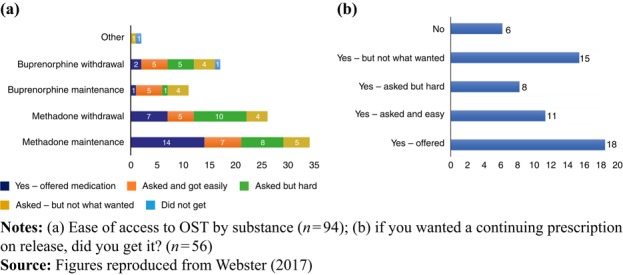
Ease of access to OST whilst in prison and to continued prescription on release

Figure 1(a) provides a breakdown of access to medication by substance, where it can be seen that respondents found it much easier to access methadone with over half (33/60=55 per cent) getting what they wanted easily and a large majority (51/60=85 per cent) achieving their goal, if sometimes after a lengthy battle. This compares with less than half (13/28=46 per cent) of those wanting buprenorphine getting what they wanted easily and almost one-third (9/28=32 per cent) not achieving their goal.

Respondents were then asked to consider the aforementioned questions with respect to whether, if they received medication in prison, they were offered a continuing prescription on release. In total, 86 survey respondents answered this question and 56 of them wanted medication on release. Figure 1(b) shows that just over half (29/56=52 per cent) were either offered medication on release or asked for it and secured it easily. However, almost two-fifths (21/56=38 per cent) either received no medication or did not get the type or as much as they wanted.

Overall, the survey showed that the choice of OST was largely limited to methadone or buprenorphine and that OST provision often differed from that requested by the individual, with methadone being the most common substitute medication provided (Figure 1(a)).

Following release from prison, the continuity of treatment on re-entering the community was variable, with some individuals finding the transition straightforward and their treatment needs met (Figure 1(b)); however, others did not receive the OST they requested or found that doses were frequently inconsistent with those received in prison or restricted to 1–3 days following their release. In some cases, no provision was made for continuity of OST after the individual’s release from detention; any disruption to treatment provision frequently resulted in relapse and a return to illicit opioid use.

Key themes from this research showed that the quality of OST provision is broadly inconsistent across the prison system and is not uniformly coordinated with community provision or with the needs of every individual receiving the service. Sometimes prison OST is appropriate for arriving inmates, and is readily available – sometimes more so than out in the community. At other times, those arriving in prison are given a lower dose of OST medication than they had been receiving, or a different medication altogether, for no obvious reason, without checks conducted for their medical or psychosocial profiles. For administrative reasons, these inappropriate dosage regimens are sometimes difficult to modify. Inmates who happen to arrive after hours can have long delays in treatment, or may not receive treatment at all, facing possible withdrawal symptoms.

Such findings contravene current government guidelines; uninterrupted treatment in custody, integrated with psychosocial care, and with clear plans for what happens on release are mainstays of the prison recommendations within the “Orange Book” (Independent Expert Working Group, 2017).

In the online survey, satisfaction with OST provision was graded as “terrible”, “poor”, “good” or “excellent”. The overall survey results showed that this experience was evenly split, with 48 per cent of respondents giving an experience of “terrible/poor” and 49 per cent stating “good/excellent” (Webster, 2017).

The findings from this survey were discussed in a focus group with experts by experience (individuals with recent experience of OST in prison). These focus group members suggested a number of key improvements including: increasing the available support for prisoners requiring OST; reducing the availability of illicit drugs, especially new psychoactive substances; providing dedicated recovery wings in prisons for those with opioid dependence; and increasing the continuity of care during the transition between prison and re-integration into the community healthcare system after release.

## Exploring existing models of OST provision in prisons: what does “good” provision look like?

An expert group consisting of individuals with extensive experience of OST in prisons from a range of perspectives (clinicians, psychosocial care providers, prison inspectors, researchers and service user representatives) was convened to develop an authoritative assessment of current practices and to make recommendations for improving OST service delivery. Details of the members of this expert group are provided at the beginning of this paper.

Following the findings of the Webster Survey (2017), the group was asked to answer the following questions:

- What is the availability of OST in prisons currently?
- How efficient is the OST service generally?
- What are the recent trends in prison OST provision?
- What is the extent of user/prisoner choice around substance, maintenance or withdrawal, and dose?
- What are the practicalities in terms of choice and diversion of medication?
- What are the challenges facing OST programmes?
- What are the important features of best practice in OST provision?

Answers were gathered by the group Chair after moderated discussion, and sorted into a consensus of current landscape observations in prison OST provision in the UK, and into recommendations for potential improvements.

Recent positive examples of best practice in the delivery of OST in a prison setting were examined. Where an overall priority was given to addressing drug use and treatment in prisons, OST treatment provision was optimised by the creation of separate recovery wings for those on OST and those not receiving substitute treatment. Once effective OST provision was in place, further steps could then be taken to address the reduction of harm, supply and demand, leading to increased benefits throughout the prisons and ultimately contributing to making the prison environment a safer place for all prisoners and staff.

An example of this effective approach cited by several expert group members was operative at a UK prison (the prison) between 2011 and 2013 (see the later section). The success of this approach was attributed to strong leadership by the prison governor, who positively influenced the entire prison staff, who were, in turn, supportive of prioritising treatment for drug addiction as a means of making improvements throughout the prison as a whole.

Undertaking such an approach requires a general culture change to ensure that prisoners requiring OST are not effectively punished by having their medication denied or delayed and where inadequacies in the provision of OST are recognised and addressed. The advantage of having a supportive prison governor to champion good OST provision is that they demonstrate what good practice looks like so the OST regimen can be driven and aligned to this view. In addition, the integration of best practice within the whole prison is essential to allow an overall understanding of the individual’s needs (e.g. why extra time with a counsellor or pain clinic may be required).

Tellingly, practice at the prison was considered to have deteriorated following the promotion and transfer of the governor who had led this approach of prioritising effective OST.

It is equally important to acknowledge what bad practice looks like and that there is a need to challenge any attitudes among prison staff that are contrary to the desire to avoid all unnecessary drug-related deaths and harms. All prison staff should be required to align with the message that recovery from problematic drug use is an achievable goal for many and that help is available to reduce harm in the meantime. Increasing the recovery rates from drug addiction is a key step towards addressing bad practice.

## New treatments and regimens for improved OST provision in prison

The expert group shared the view that one of the key factors affecting the lack of patient choice around OST medications was the concern that opiate substitutes (and other medications prescribed in prison) were routinely diverted and sold illicitly to other prisoners. Recommendations for improvements in OST provision were put forward in full knowledge of the importance of minimising the diversion of OST medication.

One suggestion for improving OST administration in prison may be alternate-day dosing, simply to reduce the queues for supervised self-administration of substitute medications. Some within the expert group questioned the practicality of this approach in the prison setting however, noting that introducing such changes would require a rigorous system to avoid any mistakes with dosing.

Alternate-day dosing has been found to be efficient (Magura *et al.*, 2009), halving the number of times a patient needs to stand in a queue. Medically, it is perfectly sound. Buprenorphine can be given on alternate days in higher doses and it requires a shorter drug-free period than methadone before induction with naltrexone for prevention of relapse (British National Formulary, 2018). There is some evidence that alternate-day treatment using multiples of the daily doses may yield better outcomes than daily dosing (Amass *et al.*, 2000). It “may suit some patients”, in the language of the Orange Book (Independent Expert Working Group, 2017). According to a study in 1998, patients overwhelmingly prefer it (Amass *et al.*, 1998). There is a shortage of literature on any operational drawbacks to alternate-day administration that may exist. The Department of Health’s (2006) “Clinical management of drug dependence in the adult prison setting” simply made no comment on it.

Prisons and other closed facilities create opportunities for transmission of infectious diseases, particularly HIV and viral hepatitis, both during detention and after release (Wirtz *et al.*, 2018; Peate, 2011). A recent eight-year study in Australia showed that, upon entry to prison, injecting drug use decreased but syringe sharing increased among injectors. Younger individuals are most likely to exhibit high-risk injecting behaviours while in prison (Cunningham *et al.*, 2018).

Thus, there is a disproportionate impact of HIV and hepatitis C (HCV) on prisoners worldwide (Sander and Murphy, 2017). Current estimates suggest that 15 per cent of all prisoners worldwide are chronically infected with HCV, and this number is even higher in regions with high rates of injecting drug use (Bielen *et al.*, 2018). Sharing of injecting drug equipment in prisons has undeniably contributed to higher prevalence of blood-borne diseases in prisoners than in the general population.

Prevention programmes specific to key populations are important, particularly for populations that are criminalised and/or may cycle in and out of prison (Wirtz *et al.*, 2018). Surprisingly few exist (Lazarus *et al.*, 2018). More research is needed on exactly how effective harm-reduction programmes are (Lazarus *et al.*, 2018). Reductions can be modest and require long-term sustained intervention coverage (Vickerman *et al.*, 2012), but there is good evidence for the effectiveness of OST as a form of harm-reduction intervention, because it reduces injecting risk behaviour. OST is associated with a reduction in the risk of both HIV and HCV acquisition (Platt *et al.*, 2017).

In addition, in a supportive environment, prison can represent a “teachable moment” for individuals who feel able to follow a detoxification programme with support from within the prison prior to release. At present, if an individual’s initial efforts to detox are unsuccessful, re-initiation onto methadone prior to release is frequently seen. This represents a negative step for the individual, which could jeopardise their efforts to detox in the future.

The current administration of OST in prisons typically involves long waiting times and queues, as well as considerable prison officer resources to supervise the consumption of substitute medications and to ensure that oral doses are taken as intended and not diverted for sale and misuse within the prison. The new formulations of controlled drugs were considered by expert group members to be positive developments that will help ensure that OST administration is as streamlined and efficient as possible. This includes the fast-dissolving wafer formulation of buprenorphine (Espranor), which has recently been approved for use across the NHS in the UK. There are other novel formulations of buprenorphine currently under review by the regulatory authorities, including long-acting depot-style injections and an implant that may prove beneficial in preventing the diversion and misuse of buprenorphine-based OST medications in prison (Bi-Mohammed *et al.*, 2017). Liquid formulations of other drugs commonly prescribed alongside opiate substitutes, such as gabapentin and diazepam have also been recently developed (Gabapentin Summary of Product Characteristics, 2019; Diazepam Summary of Product Characteristics, 2018).

## Case study: an effective prison drug-treatment strategy

This case study highlights an effective approach to tackling drugs employed at the prison between 2011 and 2013. The write-up of the experience at the prison has been developed based on the experiences of expert group members and the report from an unannounced inspection by the HM Chief Inspector of Prisons (2013).

The key characteristic of the approach at the prison was the governor’s prioritisation of tackling drugs, with an emphasis on providing excellent treatment as well as reducing supply. A whole-prison approach was taken, which was underpinned by a belief in the importance of staff–prisoner relations. This was identified by the prison inspectors:

At the heart of the prison’s success were very good staff–prisoner relationships, which were among the best we have seen in a local prison. Most prisoners told us they were treated with respect, and this was reflected both in the individual interactions we observed and well-developed consultation arrangements.

(HM Chief Inspector of Prisons, 2013)

The inspectors noted that the prison was characterised by “a generally safe and respectful environment [which] created the conditions in which prisoners could have a very good amount of time out of cell” (HM Chief Inspector of Prisons, 2013). This, in turn, helped to create a virtuous circle of continuous improvement:

Because the prison was generally safe, prisoners could spend a lot of time out of their cell. Prisoners used this opportunity to take part in activities likely to reduce the risk they would reoffend. Because prisoners felt they were making progress, that helped make the prison safer and relationships more relaxed.

(HM Chief Inspector of Prisons, 2013)

A comprehensive treatment programme was put in place for all prisoners with substance-use problems (including alcohol), a staged approach that began with their reception in prison. Prisoners were steered through detoxification or maintenance treatment in the recovery unit (D wing) and into intensive support and group work in the post-recovery unit (E wing). The inspectors highlighted the high-quality supervision and support provided by the uniformed officers in both of these wings and noted that they had received specific extra training on substance misuse.

Clinical reviews of prisoners were led by a GP specialising in substance use, and involved Integrated Drug Treatment System (IDTS) nurses and Counselling, Assessment, Referral, Advice and Throughcare (CARAT) workers. Prisoners typically stayed in the recovery unit for six weeks, where they had access to regular one-to-one support, IDTS psychosocial group work, Narcotics Anonymous and Alcoholics Anonymous (AA) groups and a good range of clinics and classes. The recovery unit also provided peer support from prisoner “recovery champions”. Similar provisions were available in the post-recovery unit, with the addition of a Self-Management and Recovery Training recovery programme for addictive behaviours. Throughout the process, prisoners who failed mandatory drug testing were subject to special support reviews. Psychosocial support was available throughout the prison.

At the time of the inspection, there were 218 prisoners receiving opiate substitution treatment, of whom 80 were on reducing detoxification doses. The emphasis on dose reduction was assessed by inspectors as “impressive” and “largely well-received by prisoners” (HM Chief Inspector of Prisons, 2013). Alcohol treatment was also integrated into the recovery-centred approach, with access to detoxification, one-to-one sessions with CARAT staff, AA and group work, including a special compulsive binge-drinkers’ group.

The success of the prison’s approach was particularly noteworthy because it was delivered in a prison that:

[…] had a notorious reputation for violence and brutality. At the time of the inspection it faced many of the typical challenges of a large, Victorian, inner-city local prison: it was chronically overcrowded, the physical condition of some parts of the prison was poor, and it held a challenging and needy population.

(HM Chief Inspector of Prisons, 2013)

The inspectors also noted that there remained work to do, and that – although impressive – the improvements were fragile. This assessment proved prophetic, because progress quickly stalled, at least partly because of the transfer of the governor. An Independent Monitoring Boards (2017) report stated that drug use at the prison was unacceptably high.

## Improved management strategies for OST provision in prisons

### OST initiation

An effective OST strategy requires clear expectations of what the affected individual is to receive when entering prison, during detention and on release. From the perspective of the prison, the completion of a full needs assessment for addiction services was found to be rarely carried out either by commissioners or providers. From the perspective of prisoners, entering the prison system can be a confusing time when a large amount of information is expected to be absorbed. Such conditions are not always conducive to effective decision making regarding the correct OST prescription.

Despite the Orange Book stating the broad expectations for treatment in prisons (Independent Expert Working Group, 2017), currently individuals cannot rely on their OST continuing while in prison. There is a need to streamline the transition into custody so that prisoners can be confident of receiving continued OST at the correct dose and understand the different regimens for detoxification or maintenance treatment so that an informed choice can be made regarding their treatment. It is important for prison services to realise that dependent opioid users being received into treatment in prison may already be experiencing withdrawal symptoms and may be preoccupied by the need to secure any form of OST; they may not be in an appropriately secure emotional state to discuss their medium- to long-term needs and wishes.

The expert group suggested that there would be considerable value in the prison service co-producing, with drug user and prisoner groups, a short national statement of basic rights for opioid-dependent prisoners in terms of what OST options will be available in prison with an emphasis on ensuring informed patient consent to different treatment regimens. It was envisaged that this statement could be displayed in the prison reception and healthcare areas.

It is also important to ensure that the correct OST dose is provided in a timely manner to avoid withdrawal symptoms that would compromise the individual’s ongoing care. Owing to the large numbers of people entering prison with OST needs, an initial goal of stabilising on methadone or buprenorphine should be set, after which they should be assessed to determine whether maintenance or reduction treatment is more appropriate, with flexibility being provided for prisoner involvement in relation to their preferred choice of dosage and treatment type.

### Transition from prison to community OST provision

Similar consideration needs to be given to continuity of treatment for those leaving prison and re-integrating into community healthcare. This is a moment of great vulnerability for the addict who is trying to recover, a period characterised by the Orange Book as “high risk” (Independent Expert Working Group, 2017). The current (positive) practice of moving people towards the end of their sentence to a prison located nearer their home often results in disruption to their OST. There is a need for ongoing assessment at all times throughout a prison stay to ensure continuity of treatment and that the correct OST regimen for maintenance or detoxification is prescribed during this transition, with the full informed consent of the patient.

Hand-in-hand with this is the importance of developing protocols and procedures to ensure that the OST provided in prison is adopted seamlessly by community healthcare providers to preserve the continuity of treatment after release. On this, the Orange Book is adamant, throughout Chapter 5, calling for professionals involved in rehabilitating prisoners “to establish effective working relationships and open channels of communication with substance misuse providers and healthcare staff in custody and in the community to ensure that treatment requirements and conditions are met and that continuity of care arrangements are supported” (Independent Expert Working Group, 2017; for the USA, see Patel *et al.*, 2014). This clearly necessitates communication between OST providers, probation services and housing provision services upon the individual’s release, to avoid any delays in receiving OST due to errors in assigning an address and difficulties with housing arrangements. It is acknowledged that prisoners are often not released to secure accommodation and that care needs to be taken to ensure that community prescribing arrangements are compatible with both the individual’s immediate housing situation on release and any likely changes in the short to medium term.

The timing of release also needs to be taken into consideration to avoid gaps in treatment which may occur following a Friday release when prescriptions may not be filled until the following Monday (Independent Expert Working Group, 2017). Care arrangements need to be coordinated so that housing provision, OST and psychosocial support are not disrupted on the individual’s release from the prison. There are benefits to the wider community in terms of crime reduction following a positive experience of leaving prison, with the individual’s needs for OST provision having been addressed as part of a coordinated release process.

In the community primary care setting, the FP10 prescription system aims to ensure that prescriptions are made available to prison healthcare services so that a seamless transfer of prescribing care is made between prisons and the community upon release of inmates. A prescription is provided on prisoner release, often for several doses of OST, but for not more than one to three days’ duration. This system is not currently in universal use and, in the absence of any published feasibility studies, the authors feel it should remain this way. Managing several doses for the transition out of prison would need coordination in relation to home visits to ensure the safe storage of medication within the home, for reasons of child protection. Access to prescribing services in the community can be difficult to organise, particularly if a newly released prisoner misses an initial appointment, but the consideration of a right to emergency access should be made in the interest of the individual’s health and crime reduction in the community. The prisoner’s physician records should be checked in advance of release to ensure that the individual has confirmed access to primary care on release.

Another key element of release planning is the provision of advice and information about the increased risk of death from overdose that comes from reduced opioid tolerance (including the provision of relevant information to the individual’s family and friends) and the routine provision of naloxone, which rapidly reverses the effects of heroin or methadone – the most lethal of which being the way they cause respiratory depression, the factor most closely associated with death by overdose. The expert group’s experience was that the availability of naloxone for released prisoners varied considerably between different prisons in England and Wales.

The period following release from prison is a time of extraordinarily high mortality (Strang *et al.*, 2013). In Scotland, the provision of overdose-prevention training and the supply of take-home naloxone kits to people on release from prison has been established as part of a nationally funded programme (the first in the world) since 2011. In this time, 12,000 kits have been issued from prisons across Scotland. A recently published evaluation of this programme demonstrated a 36 per cent reduction in opioid-related deaths in the four weeks following release from prison (Bird *et al.*, 2016; Strang *et al.*, 2014). Worldwide, take-home naloxone has been available for 20 years now (McDonald *et al.*, 2017; Dettmer *et al.*, 2001). Provision for its use has widened, with public buy-in (McDonald *et al.*, 2017). It needs to be widened even more, as the death toll due to overdosing continues to rise (McDonald *et al.*, 2017). The logistics of this are currently under investigation in the UK (Meade *et al.*, 2018).

There is much left to learn, and study should continue. It is now established that prison-based substitution therapy reduces drug use and injection in penal institutions and brings down drugs charges and re-admission rates (Stallwitz and Stöver, 2007). This is as true for women offenders as for men (Farrell-MacDonald *et al.*, 2014). The numbers are not always enormous. In one recent study, by way of example, within 12 months of release from prison, 58 per cent of heroin users who did not receive OST had been re-incarcerated, compared with 41 per cent of those who did receive OST (Stöver and Michels, 2010).The effectiveness of methadone therapy measured in terms of treatment retention and recidivism varies from study to study, from as little as 20 per cent to as much as 70 per cent (Crnić, 2017; for the methodological difficulties in conducting studies in prison settings, see de Andrade *et al.*, 2018). Numerous clinical studies are now investigating the reasons for its effectiveness, both on the underlying clinical symptoms of addiction and on social and health implications. Interestingly, being allowed to continue in prison an OST regimen begun before arrest may also have a beneficial effect on eventual recidivism rates (Westerberg *et al.*, 2016). Length of care and consistency, whatever else is true, do appear critical (Westerberg *et al.*, 2016).

### Integrating clinical and psychosocial care in prisons

In order for OST provision to improve, the expert group shared the view that an integrated approach that addresses all aspects of healthcare during detention is needed (Sander *et al.*, 2016). To achieve this, integration between clinical and psychosocial care is essential. Often these services are commissioned separately; where this is the case, it is important that all drug-treatment staff are co-located so that the same account of patient needs is heard by all members of the care team and a coordinated treatment/care plan can be implemented and managed.

Currently, it is not unusual for a named drug worker to be assigned to a prisoner entering prison. However, in some cases, a named clinical nurse is also available to allow the development of a joint care plan. This care plan should then be reviewed with the full care team present so that the patient’s clinical and psychosocial care can be assessed and ongoing plans can be formulated involving all the relevant aspects of an individual’s care. Such integrated care would allow the prisoner a clearer view of their treatment as a whole and enable them to make better-informed decisions regarding their future care.

Making decisions without such information may fuel the prescription of higher-than-needed maintenance doses of methadone as clinical staff have insufficient information regarding the psychosocial welfare of the service user; higher doses are then justified as there is a lack of psychological support for those on lower or reducing doses of OST. A good care model for OST would have clinical and psychosocial care fully integrated to instil confidence that all aspects of care are being covered. It is envisaged that this would also include care plans for additional health issues, such as general self-care and management of smoking cessation, together with the integration of social services with OST provision within the prison setting.

## Diverse prison populations need tailored provision of OST

Optimising the care model for provision of OST in prison will need to take into account several diverse patient populations. Recent policy directives within the Scottish Prison Service point out that many health promotion activities target the general prison population and may not address the needs of minority groups. The framework argues the importance of using an impact assessment (such as the “Health Inequalities Impact Assessment”) on all new prison policies “to understand the impact on health and wellbeing on all prisoners but especially of the minority groups”. The Service defines minority groups to include (among others) older prisoners, prisoners with any kind of disability, women and prisoners of minority ethnic origin (Scottish Prison Service, 2014). Brigadier Hugh Monro, HM Chief Inspector of Prisons for Scotland, stated that although much has improved in prison buildings in recent years, rehabilitation is still a major issue. He says there needs to be a strategic shift in the way prisoners are rehabilitated, particularly women prisoners, who are simply not being given enough to do during the day, especially with regard to work and education opportunities (HM Chief Inspector of Prisons, 2012–2013). Occasional initiatives in the UK, sometimes outside the realms of work and education, show promise of enriching inmates’ lives and making them more amenable to rehabilitation. One such is the “Good Vibrations” arts project for older offenders, whose result was perceptible increase in insight and reflection in individual prisoners and a stronger cohesion in groups of prisoners (Wilkinson and Caulfield, 2017). Broadly speaking, though, sustained and successful efforts at meaningful rehabilitation strategies are difficult to find. It is within this reality that policies for provision of OST reside.

### The older offender

There is an increasing population of ageing incarcerated drug users (PPO, 2017), with additional comorbidities, poly drug use and general additional health issues that affect their OST needs. Psychological disorders vary with age (O’Hara *et al.*, 2016). Some severe functional impairment has been shown to decrease as opioid maintenance age increases, but depression is routinely higher in older people (O’Hara *et al.*, 2016). In addition, somatic comorbidity, particularly the incidence of liver disease, also increases with age. Social isolation and the despair over reconnecting with family after years of neglect are other documentable features of older patients in recovery (Gaulen, 2017). Indeed, many of the older population can be expected to have very poor social networks. Factors like these underline the need for coordinated care within prisons and the need for continued, consistent care in the community healthcare system following release. However, care has not been forthcoming. In the past eight years, the Prison Reform Trust has found that the majority of prisons have nothing specific in place to support the resettlement needs of older people, and that older people feel that planning for their release is inadequate. The government introduced radical changes to support people on release from prison with the Care Act 2014, with its wide-ranging implications for the care of older people (Cornish *et al.*, 2016). It remains to be seen what effect this will have.

### The mentally ill

In a survey of psychiatric morbidity in prisons in England and Wales, 97 per cent of sentenced drug-dependent men had at least one co-occurring mental health condition and 77 per cent had at least two such conditions (Singleton *et al.*, 1999). Hence, there is likely to be a widespread occurrence of prisoners requiring treatment for both their opiate use and for their mental health concerns. The clearer coordination of treatment and any relevant treatment sequencing is needed to avoid individuals in prison being sent back and forth between mental health support and OST supervision, with each requiring the other to be addressed first. Ongoing care for the ill after release has been limited. Despite this, it has been shown in trials that opiate substitution treatments reduce substance misuse relapse and possibly reoffending (Fazel *et al.*, 2016).

### Women

Women in prison present with unique requirements for OST. Data suggest that incarcerated women are more likely than men to inject opioids, are more likely to be addicted to multiple substances and are at an increased risk of post-release overdose. They have higher rates of psychiatric comorbidities than men or non-incarcerated women and are more likely than men to have chronic or communicable diseases (including HIV and HCV). Women offenders have higher rates of trauma and are more likely than men to be victims of staff misconduct while incarcerated. Nearly two-thirds of women in prison have dependent children at home. They are less likely than men to commit violent crimes, but more likely to commit other drug-related offences (Evans, 2015). These realities align with women’s existing, often complex physical and mental health concerns, and additional considerations need to be taken into account if there are children in their care or in the care of local services. The coordination between prison and community services is especially important where assessments may be needed of the women’s home environments if OST medication is to be stored within the home and children may be present. Unfortunately, the evidence remains slim that the introduction of women-specific policies and support programmes has had a great impact on womens’ post-release success (Carlton and Segrave, 2016).

### Ethnic and cultural minority populations

Similarly, improvements in cultural competence in the provision of addiction treatment within the British Black, Asian and minority ethnic population are needed; the survey conducted for this paper (Webster, 2017) reported that people in this patient population find OST harder to access although treatment provision should strive for consistency for all individuals in every setting. The Orange Book does encourage particular consideration of minority ethnic groups in treatment, to deliver OST in culturally sensitive ways wherever possible (Independent Expert Working Group, 2017). Research into how well this is being done is only beginning. Likewise, studies on institutional provision for after-prison care that is specifically for minorities are hard to find. One study noted the considerable difficulty implementing a service: a methadone maintenance programme in an indigenous community in Canada had difficulty in uptake because the intervention was misunderstood at first and stigmatised (Landry *et al.*, 2016).

## Educational needs for healthcare providers and administration of OST

Many GPs lack experience with the administration of OST within the prison environment and the expert group advocated the provision of a key drug worker for GPs (analogous to the “shared care” model prevalent in the community) to be a source of reliable information when they are unsure of any specialised knowledge about opioid use, misuse or treatment.

Aligned with this is the assessment of the availability of drugs within a particular prison. The examination of specific local markets may give very different pictures (including from both the dispensing and service-user perspectives); assessment should focus on which drugs are available and the extent of the diversion of OST medications within a particular prison. It is clear from feedback that these drug markets can change very quickly in relation to supply and demand and the desire to use more in prison due to the circumstances of incarceration. Prison drug markets have recently been complicated by the availability of large amounts of different new psychoactive substances (User Voice, 2016).

The expert group also agreed that introducing more structured days and more purposeful activities for prison inmates would offset the inherent boredom and long queues for treatment administration, resulting in greater adherence and compliance with OST regimens. Aligned with this is the lack of OST integration within the prisons’ wider healthcare services – it is suggested that by working more closely together, integrated healthcare services, including those given by visiting GPs, would help improve engagement with OST provision overall. However, it was noted that at present there is some tension between clinicians dealing with the administration of treatment for OST and those dealing with the rest of prison healthcare.

## Summary

Barriers to effective management of OST in prisons have recently been identified (Independent Expert Working Group, 2017). These barriers include a lack of coordination of care between healthcare providers in prison, resulting in the disrupted management of addiction. The integration of clinical and psychosocial care in prisons would enable a joint care plan for each individual with opioid dependence to be developed and modified as required.

Improvements in the overall coordination of OST administration would facilitate the inclusion of strategies to further streamline this process for the benefit of individuals in prison and prison staff alike. Improvements would include clearer information being made available for opioid-dependent individuals about the range of OST medications available and whether these are prescribed on a maintenance or reduction basis. Further study is needed into the viability and desirability of alternate-day dosing for supervised self-administration of OST medications.

Prioritising effective management strategies for drug addiction in prisons and championing by senior prison managers would foster a more supportive environment for the successful delivery of maintenance or detoxification OST as needed.

Training prison inmates with experience of OST to provide peer support and advocacy to individuals seeking OST on their reception into prison is an initiative that was also recommended for further exploration by the expert group.

The expert group was convened before the publication of the 2017 *Drug Strategy* (HM Government, 2017). That strategy sets out a number of key actions to address drug use in prison that are consonant with the recommendations in this paper, including:

- governors working in partnership with health commissioners to co-commission integrated and patient-focussed drug treatment programmes;
- improving continuity of care with community services;
- reassessing the substance misuse treatment pathway for prisoners and how these services, including peer support, meet the treatment and recovery needs of offenders;
- more highly trained prison officers playing a bigger role in the provision of services while building more constructive and relevant relationships with offenders; and
- developing options to address the misuse of prescribed medicines more effectively.

To go along with this, we propose the following expedients:

- establish a clear OST strategy between care team and prisoner at the moment of arrival, with careful note of the individual’s specific needs;
- ensure that the OST is prompt, reliable and can be adjusted;
- establish clear protocols for transition to the community on release, with no gaps in care, and no changes, and with adequate safeguards against overdose; and
- integrate clinical and psychosocial care, using a full-team care plan that regards the prisoner as part of the team.
